# Parameterising Translational Feedback Models of Autoregulatory RNA-Binding Proteins in *Saccharomyces cerevisiae*

**DOI:** 10.3390/microorganisms10020340

**Published:** 2022-02-01

**Authors:** Michael Clarke-Whittet, Andrea Rocco, André P. Gerber

**Affiliations:** 1 Leverhulme Quantum Biology Doctoral Training Centre, University of Surrey, Guildford GU2 7XH, UK; m.clarke-whittet@surrey.ac.uk; 2 Department of Microbial Sciences, Faculty of Health and Medical Sciences, University of Surrey, Guildford GU2 7XH, UK; 3 Department of Physics, Faculty of Engineering and Physical Sciences, University of Surrey, Guildford GU2 7XH, UK

**Keywords:** kinetic modelling, RNA-binding proteins, feedback, autoregulation, yeast

## Abstract

Post-transcriptional gene regulation is driven by RNA-binding proteins (RBPs). Recent global approaches suggest widespread autoregulation of RBPs through binding to their own mRNA; however, little is known about the regulatory impact and quantitative models remain elusive. By integration of several independent kinetic parameters and abundance data, we modelled autoregulatory feedback loops for six canonical and non-canonical RBPs from the yeast *Saccharomyces cerevisiae*, namely Hrb1p, Hek2/Khd1p, Ski2p, Npl3p, Pfk2p, and Map1p. By numerically solving ordinary differential equations, we compared non-feedback models with models that considered the RPBs as post-transcriptional activators/repressors of their own expression. While our results highlight a substantial gap between predicted protein output and experimentally determined protein abundances applying a no-feedback model, addition of positive feedback loops are surprisingly versatile and can improve predictions towards experimentally determined protein levels, whereas negative feedbacks are particularly sensitive to cooperativity. Our data suggests that introduction of feedback loops supported by real data can improve models of post-transcriptional gene expression.

## 1. Introduction

RNA-binding proteins (RBPs) play an essential role in the post-transcriptional control of gene expression with imminent functions in controlling cell homeostasis. In eukaryotic cells, hundreds of different RBPs are present, and bind to RNA in a combinatorial fashion, forming ribonucleoprotein (RNP) complexes that implement biological regulation [1]. RBPs binding to mRNAs control mRNA capping, processing, localisation, translation, and eventual decay. Thus, information contained in proteins, such as type, state, and copy numbers, can be transmitted back to the mRNA level for fate determination, and vice versa. This is possibly seen most pervasively in translation initiation, as a major point of contact which every mRNAs must pass for protein expression.

Numerous proteins integrate cellular homeostatic and sensory signals, by gatekeeping of mRNA access for translation on a global or specific basis [2]. Eukaryotic translation initiation is considered to be the rate limiting step in protein expression and is dependent on the activity of translation initiation factors (eIFs), providing tight control of translation. For instance, the activity of the cap-binding protein eIF4E, which recognises the mRNA 5′ m^7^G cap, can be enhanced by phosphorylation or inhibited by binding of inhibitory proteins, such as eIF4E binding proteins (4E-BPs), thereby competing for interaction with eIF4G to form the eIF4F initiation complex [3,4,5]. The phosphorylation of 4E-BP, which alleviates interaction with eIF4E, is catalysed by the mTOR kinase, which in turn can be repressed by the nutrient-sensitive protein, raptor [6]. Thus, cellular protein synthesis is coupled to nutrient sensing by tuning the activity initiation factors, allowing single cells to alter gene expression regimes on a global level.

Other RBPs, however, interact with more defined elements in mRNAs, enabling more specific points of control. For example, eIF4A, a DEAD box containing RNA helicase, unwinds RNA structures in the 5′ untranslated regions (UTRs) of mRNAs and comprises the third member of the heterotrimeric eIF4F complex; while the poly(A)-binding protein (PAB) that contains several RNA-recognition motifs (RRMs) interacts with the poly(A) tail at the 3′ end of eukaryotic mRNAs, and facilitates interaction with eIF4G to promote translation initiation [7,8]. Hence, these ‘generalist’ RBPs discriminate between coding and non-coding and recognize mRNA-specific features, such as structural elements and poly(A) tail lengths, for selective control of translation.

The control of particular mRNAs can be implemented through highly specific RBPs, that often recognize a structural or sequence code in the mRNAs. For example, the Puf protein family contain specific Pumilio homology domains (Pum-HD), which confer specific interactions with 8–10 nucleotide long sequence elements in their mRNAs targets, particularly located in 3′ UTRs [9]. RBP immunoprecipitation microarray (RIP-chip) analysis revealed that Puf3p preferentially interacts with mRNAs encoding mitochondrial proteins, whereas Puf4p and Puf5p (Mpt5p) bound mRNAs code for nuclear proteins [9]. This suggests an overarching coordination of functionally related mRNAs by RBPs, forming so-called post-transcriptional operons or RNA regulons [10,11].

Feedback is a well-established way for biological systems to achieve multistable and oscillating responses, often through combination of several interacting feedback loops [12]. Transcriptional feedback, in conjunction with post-transcriptional control, frames highly interconnected regulatory gene expression networks that can lead to periodic biological systems like the circadian rhythm [13,14]. It also equips microbes with another regulatory tool to sense and quickly adapt to changing environmental conditions [15]. A special case of feedback, autoregulation, can arise in post-transcriptional control when an mRNA’s expression is regulated by its RBP product. Autoregulation is a low-complexity feedback loop allowing an RBP to regulate itself without a second regulatory molecule [16]. As such, it is a rather economical system, which lends itself well to characterisation by modelling.

Despite its importance, only a few examples for autoregulation have been characterised in the budding yeast *Saccharomyces cerevisiae*, a well-annotated single-celled eukaryote, and a suitable system for modelling. For example, a recent study used a GFP tagged library [17] to measure expression of GFP tagged ribosomal proteins (RPs) upon overexpression of individual untagged RP genes [18]. As the untagged gene was expressed, the abundance of the tagged version decreased for several RPs, indicating autoregulation [18]. Thereby, changes in protein abundance were responsive to sequences located in corresponding 5′ UTRs of mRNAs, demonstrated with reporter constructs bearing respective 5′ UTRs fused to GFP. Another example concerns the aspartyl-tRNA synthetase Dps1p (AspRS), which interacts with its own mRNA, seemingly to achieve homeostasis by introducing negative feedback regulation [19]. Recently, translational autoregulatory feedback loops were reported for several tRNA synthetases; among them histidyl-tRNA synthetase (HisRS), which binds to its own mRNA in a region predicted to fold into a tRNA His anticodon-like structure and controls translation according to tRNA demands [20].

The mRNA targets for 69 yeast RBPs have been previously compiled from RBP immunoprecipitation chip (RIP-chip) analysis; 26 of those RBPs were found associated with their own mRNA [21]. This suggests potential for an autoregulatory feedback function, although whether these putative feedback loops would have any effect on the expression dynamics is not known. Their effect, however, may be explored by modelling the expression from transcription to translation, considering relevant rate constants with and without a feedback loop. Here, we explored this issue by building translational kinetic feedback loops, parameterised from a variety of *S. cerevisiae* studies. The kinetic models, similar in principle to transcriptional modelling or the translational feedback model proposed by Tyng and Kellman [22], were parameterised and explored to give protein abundance predictions for a selection of putative autoregulatory RBPs. These were assessed by comparison to a measured protein abundance benchmark originated from a thorough review of protein absolute abundances in yeast [23].

## 2. Materials and Methods

### 2.1. Selection of Potential Autoregulatory RBPs

A list of 26 potential autoregulatory RBPs was extracted from [21]. Parameters *k*_1–4_ as well as protein and mRNA levels were compiled for each RBP. Complete sets of data could be retrieved for six potentially autoregulatory RBPs, which includes four canonical RBPs (Hrb1, Hek2/Kdh1p, Ski2p, Npl3p) and two mRNA-binding metabolic enzymes (Pfk2p, Map1p) (Table 1). In brief, Hrb1p is a poly(A) binding protein involved in mRNA export [24]. Hek2p regulates translation of anaphase-associated *ASH1* mRNA, by repressing *ASH1* expression in the mother, but not the daughter cell during cell division [25]. Ski2p is a putative RNA helicase of the yeast mRNA 3′ degradation complex, acting along with Ski3p and two Ski8p subunits [26]. Npl3p is a multi-functional protein that shuttles between the nucleus and cytoplasm and is involved in the regulation of Pol II transcriptional elongation and termination, splicing and mRNA export [27]. Npl3p also seems to link histone ubiquitination to translation initiation interacting both with Bre1p and eIF4G [28]. Pfk2p is a subunit of the hetero-octameric phosphofructokinase complex in the glycolytic cascade [29]. Map1p, like Map2p, cleaves the terminal methionine (arising from the translational start codon) from newly synthesised peptides in yeast [30]. Pfk2p and Map1p are so-called ‘unconventional’ RBPs, initially identified by in vitro RNA-binding studies and confirmed in vivo with RNA interactome capture (RIC) [31,32].

### 2.2. Data Acquisition

The transcription rate (*k*_1_) was taken as an average from a genomic run-on (GRO) assay [33]. Specifically, *S. cerevisiae* (strain BY4741) cells were grown in yeast-peptone-dextrose (YPD) media at 28 °C to exponential phase, and radiolabelled nucleotides were added for a short time for incorporation into nascent mRNAs. The density of incorporated moieties was used to calculate the specific mRNA synthesis rate for each gene [33]. Of note, BY4741 (*MAT*a *his3Δ1 leu2Δ0 met15Δ0 ura3Δ0*) is derived from the widely studied S288C strain [34].

mRNA degradation rate constants (*k*_2_) were taken from reported half-lives measured by deactivating transcription using a heat-sensitive RNA polymerase II mutant [35]. *S. cerevisiae* cells (*MAT*a *ura3-52 his4-539 rpbl-l*), which express a temperature-sensitive RNA polymerase II [36], were grown in rich YPD medium at 24 °C until exponential phase where the temperature was shifted rapidly to 37 °C to deplete Pol II-dependent transcription. Subsequently, the degradation of mRNA abundance was measured over time [35]. We calculated degradation rate constants as *k*_2_ = ln(2)/*t*_1/2_ from the reported half-life (*t*_1/2_) for each mRNA.

Protein synthesis rate constants (*k*_3_) were compiled from pSILAC data, where incorporation of heavy and light isotopes in proteins identified by mass-spectrometry gives the actual synthesis rate of particular proteins similar to the GRO assay [37]. BY4741 *S. cerevisiae* cells were grown to early exponential phase at 30 °C in synthetic complete (SC) media and further grown to late exponential phase in light lysine hydrochloride SC as part of the pSILAC experiment.

Protein degradation rate constants (*k*_4_) were taken from half-life measurement in translation-inhibited *S. cerevisiae* [38]. Tandem affinity purification (TAP) tagged *S. cerevisiae* strains [39] were grown in rich YPD medium to exponential phase and cycloheximide was added subsequently which prevents translational elongation. Each protein level was determined only by its degradation in the absence of any new translation for the duration of the cycloheximide exposure.

The protein abundances (*P_ss_*) were extracted from the unified protein abundance dataset, which based abundance measurements on mass-spectrometry quantification and normalised data across various individual studies [23]. Mean mRNA abundance (*m_ss_*) was integrated from absolute competition-PCR quantification from S288C *S. cerevisiae* grown in rich YPD medium to exponential phase at 30 °C [40].

Parameters for 5854 yeast proteins were compiled (bounded by the protein quantification data set [23]). The full dataset is available in the Supplementary Materials Table S1. Of those, 607 protein entries were complete for *k*_1–4_, *P_ss_*, and *m_ss_*. The *k*_3_ translation rate dataset was the most limited, including data for 1106 proteins [37].

### 2.3. Acquisition and Conversion of Single-Cell Association/Dissociation Constants

Dissociation constants (*K_d_*) were gathered from previously reported affinities of proteins to its respective RNA-binding sites: Hrb1p, *K_d_* = 3 μM [41]; Hek2p, *K_d_* = 90 nM [42]; Ski2p, *K_d_* = 500 nM [43]; Npl3p, *K_d_* = 2.5 nM [44]. As *k*_2–4_ are reported in 1/min, it was necessary to convert respective dissociation constants into molecules per cell. This was achieved by multiplying each by Avogadro’s number (6.02 × 10^23^ molecules mol^−1^) and the average volume of a haploid *S. cerevisiae* cell (assumed as a sphere with a diameter of 4 μM (by V= 4/3(πr^3^), so V = 33.5 fL) [45]). Likewise, the original *K_a_* was taken as the inverse of the converted *K_d_* (*K_a_* = 1/*K_d_*) and so has reciprocal units (cells per molecule, Table 1).

Experimentally determined dissociation constants for Pfk2p and Map1p are not known. Therefore, respective values were predicted by solving the feedback models (Equations (2) and (3)) for *K_a_* and *K_d_* using the parameters gathered in Table 1. Likewise, feedback models could be solved for Hrb1p and Npl3p.

Feedback models with cooperative binding (Hill coefficient *n* > 1) assumes protein–protein complex formation (i.e., *n* = 2 relates to the formation of a protein dimer). Since these respective rate constants of protein multimerisation are experimentally uncharacterized, we considered a complexing ratio of 1/1 (1 M) in a first instance, meaning the protein in complex is present at equal numbers as the uncomplexed protein. This complex constant constitutes another dissociation constant describing the dynamics of the protein homomer (opposed to *K_d_*, which here describes the protein-RNA complex formation). In addition—and to evaluate the sensitivity of the model—we considered the complex to be more abundant than the uncomplexed protein. Therefore, we chose an arbitrary multimerisation constant of 1 × 10^−6^ (corresponding to 1 µM), which was applied by dividing respective *K_a_* and *K_d_* by one million. 1 × 10^−6^ could be a realistic value given the scale of the reported *K_d_* values (from 2.5 nM to 3 µM) and without relying on a stronger value that may be overly optimistic and could limit the conclusions which may be drawn.

## 3. Results

### 3.1. Modelling RBP Gene Expression without Feedback Is a Poor Predictor of the Steady State Protein Abundance

**Figure 1:**
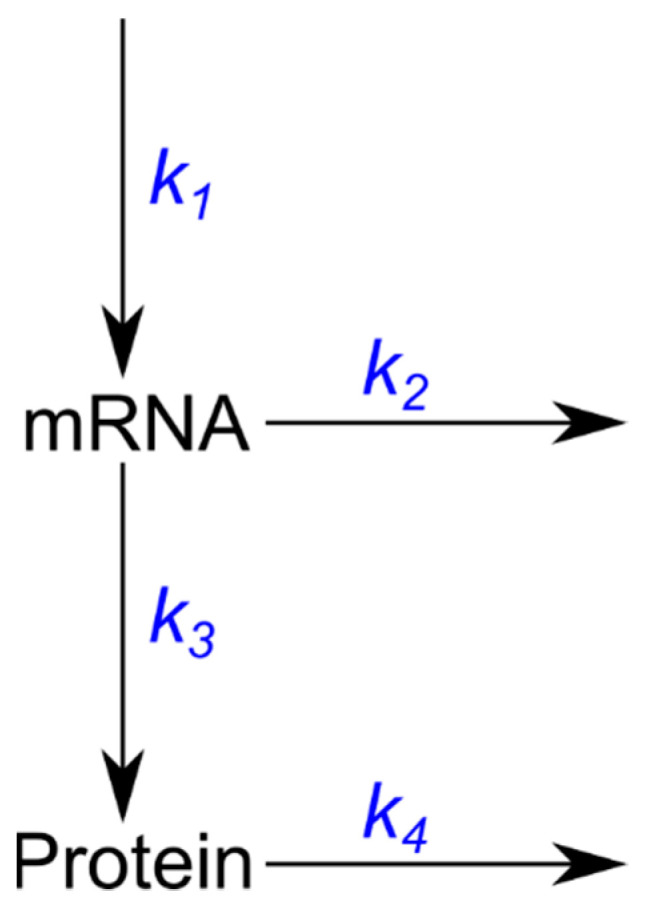
Model of gene expression based on two consecutive independent steps of transcription and translation.

We first considered the two-stage standard model describing transcriptional and translational dynamics without any regulatory feedback loops (Figure 1).

In this simple model,$\;k_{1}$ and $k_{3}$ correspond to transcription rates and translation rate constants, and $k_{2}$ and $k_{4}$ to degradation rate constants of mRNAs and proteins, respectively. This model can be represented mathematically as a kinetic model in terms of ordinary differential equations (ODEs) as follows:

(1) $$\{\begin{array}{c} \frac{dm}{dt}=k_{1}-k_{2}m\\ \;\;\;\frac{dP}{dt}=k_{3}m-k_{4}P \end{array}\;\;$$

where the dynamical variables *m* = *m*_(*t*)_ and *P* = *P*_(*t*)_ represent mRNA and protein copy numbers.

**Figure 2:**
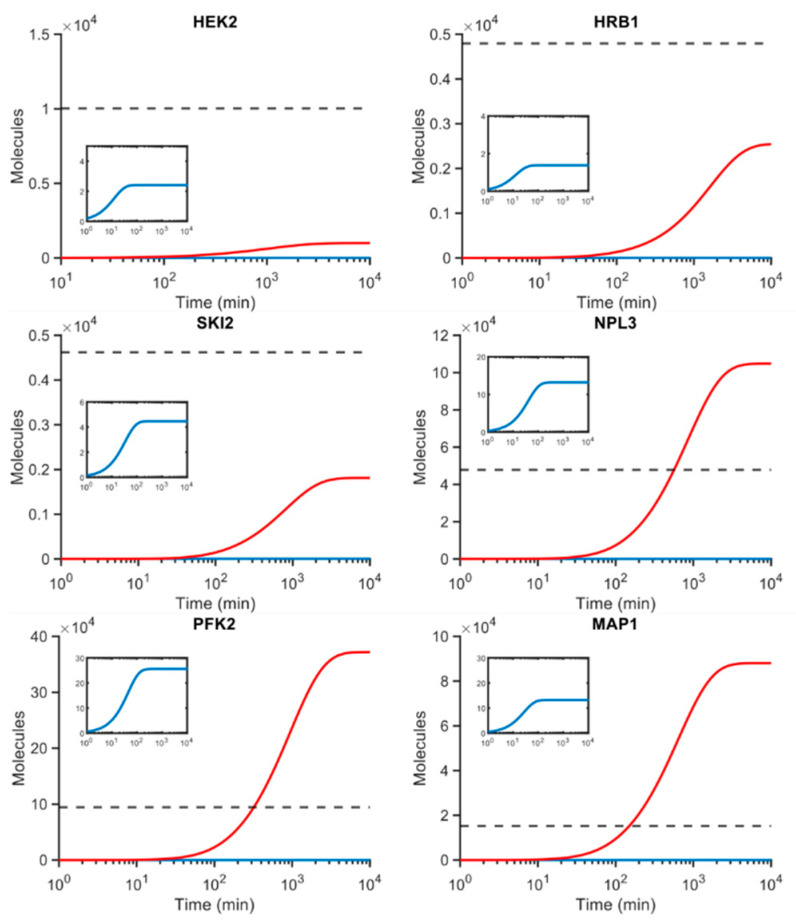
Simulated gene expression of putative autoregulatory RBPs (red) without feedback. The mRNA abundances (blue, inset) are shown initialised from 0 and evolved to the steady state of the model. The dashed grey line denotes the relevant measured protein abundance (*P_ss_*, Table 1). The mRNA abundance is markedly lower than protein abundance, and come to its steady state quickly (inset, units are copies min^−1^). Note the copy numbers scale (*y*-axis) is given in tens of thousands of molecules (10^4^); the time (*x*-axis) is in log scale.

The solution of Model (1), obtained using values reported in Table 1, is shown in Figure 2. Time traces of mRNA and protein abundances approach the steady state after around ten thousand minutes. The system being modelled is assumed to be at the steady state and initial abundances of mRNA and protein have been set to zero copies.

The models considering *HRB1*, *HEK2*, and *SKI2* predict two to five-fold lower final steady state levels than measured (‘benchmark’) average protein abundances. Specifically, Hrb1p is predicted to have a protein abundance of 2543 protein copies per cell, while the known abundance is 4794; Ski2p is predicted to have 1811 proteins, while the benchmark is 4623; and Hek2p is predicted at 997, while the target is 10,029 (Figure 2). Conversely, it overpredicts the steady state abundance levels of Npl3p, Pfk2p, and Map1p by 2–4 fold. Npl3p reaches a predicted abundance of 104,800 molecules per cell, as compared to the benchmark of 47,816; Pfk2p is predicted at 371,960, while the benchmark is 94,449, and Map1p is predicted to have 88,001 copies, while it is expected to have 15,176. This suggests that, with the parameter values given in Table 1, the topology considered in Model 1 is unable to correctly reproduce estimates for steady states values (Figure 2).

### 3.2. Gene Expression and Steady State Predictions Can Be Tuned Using Translational Feedback Loops

**Figure 3:**
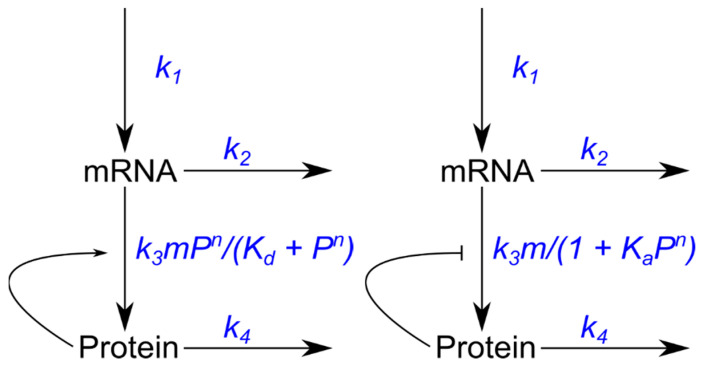
Models of gene expression with translational autoregulation. Left: positive feedback loop. Right: negative feedback loop. The synthesized protein interacts with the translation rate $k_{3},$ effectively increasing or decreasing the pool of mRNA molecules available for translation.

We next considered positive and negative feedback loops of the synthesized protein on its own overall translation rate (Figure 3).

These new regulatory systems were built based on previously described gene expression regulatory feedback models [22,46]. The feedback loops can be described with two ODEs. Equation (2) represents the negative feedback of the protein on its own translational rate $\;k_{3}$ in terms of a repressive Hill function:

(2) $$\{\begin{array}{l} \frac{dm}{dt}=k_{1}-k_{2}m\\ \frac{dP}{dt}=\frac{\;k_{3}m}{1\;+\;K_{a}P^{n}}-k_{4}P \end{array}$$

Equation (3) represents instead the case of positive post-transcriptional feedback:

(3) $$\{\begin{array}{l} \frac{dm}{dt}=k_{1}-k_{2}m\\ \frac{dP}{dt}=\frac{\;k_{3}mP^{n}}{K_{d}\;+\;P^{n}}-k_{4}P \end{array}$$

The feedback dynamics are specified in terms of the association or dissociation constant ($K_{a}$ or $K_{d}$), representing the strength of the feedback. When *n* = 1, cooperativity is not a feature of these feedback loops, which then exhibit Michaelis–Menten dynamics. If *n* > 1, positive cooperativity was introduced in the model in the form of a protein complex (i.e., as a dimer when *n* = 2 or as a tetramer when *n* = 4).

**Figure 4:**
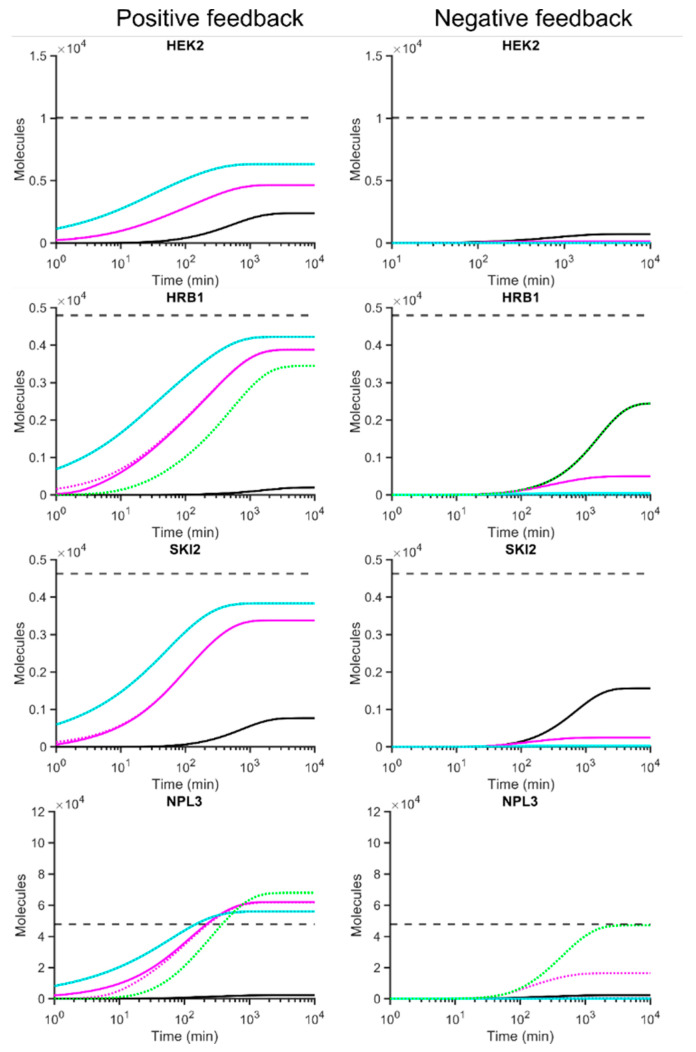
Simulated gene expression with positive/negative translational feedback loops for *HEK2*, *HRB1*, *SKI2*, and *NPL3*. Positive feedback is indicated to the left, negative feedback to the right. The dashed grey line indicates the experimentally determined (benchmark) protein abundance (*P_ss_*, Table 1). Solid black line represents each feedback with Hill coefficient of 1. The solid violet line considers Hill coefficient of 2 with a protein complexing constant of 1 M; the dotted violet line assumes a protein complex formation constant of 1 μM. A Hill coefficient of 4 with protein complexing constant of 1 M is indicated with the solid cyan line, a complexing constant of 1 μM as dotted cyan line. The calculated ‘best-case’ *K_a_* and *K_d_* for *HRB1* and *NPL3*—feedback models where *n* = 1 are shown in dashed green lines (not shown for *HEK2* and *SKI2* as these returned negative values, which are not realistic).

The three parameters $K_{a}\;\mathrm{or}\;K_{d}$ and *n* are required for the full parameterisation of the feedback dynamics. Hill coefficients of *n* = 2 and *n* = 4 were also tested with the assumption of the hypothetical protein complexing constants of either a 1:1 ratio or 1 million:1 protein complex vs. unbound protein (see Section 2.3) We solved these numerically to find their steady states as shown in Figure 4.

### 3.3. Co-Operative Positive Translational Feedback Simulations Can Predict More Accurate Steady State Protein Abundances Than Models without Feedback

We first considered binding of the four RBPs with experimentally determined *K_a_* and *K_d_* values as independent monomers (i.e., Hill coefficient, *n* = 1) (Figure 4). The introduction of the positive feedback model increased the final abundance for Hek2p (2383 proteins) as compared to the non-feedback models (997 proteins), though it was still below the experimentally determined benchmark levels (10,000 proteins). Conversely, the other three RBPs (Hrb1p, Ski2p and Npl3p) reached lower final protein abundances than no-feedback models, which seems counterintuitive, as one might expect positive feedback to accelerate protein production, as seen for Hek2p. For example, the no-feedback model for Npl3p predicted 104,800 proteins at steady-state, while introduction of the positive feedback cases reduced levels to 70,763 proteins, which is relatively close to benchmark protein levels (Figure 4).

Interestingly, the introduction of cooperativity through the Hill coefficient (*n* = 2, 4) increased the predicted steady-state levels for Hek2p, Hrb1p and Ski2p quite dramatically, leading to better approximation of experimentally determined benchmark protein levels (Figure 4). While such positive effects were observed for said three RBPs, likewise introduction of cooperativity reduced steady state levels of Npl3p, thereby leading to a better approximation of benchmark protein levels. Thus, in all cases, consideration of the Hill coefficient shifted the predicted steady state protein levels towards the given benchmark levels. Finally, we found that the dissociation constant for protein complex formation had only a minor influence in projected final steady protein levels: considering either 1 M or 10 µM dissociations constants did not greatly affect dynamic model projections. However, slight differences in early kinetics for Npl3p were observed, and thus, complex formation kinetics may mostly affect transient dynamics, but hardly final steady state.

### 3.4. Negative Translational Feedback Is Highly Repressive at Biologically Relevant Scales

Considering experimentally determined *K_a_* and *K_d_* values, projection of negative translational feedback models with monomeric proteins (*n* = 1) was highly inhibitory for the expression of Hek2p and Npl3p. It further reduced the final abundance for Hek2p (715 proteins) as compared to the non-feedback models (997 proteins). Likewise, Npl3p expression was highly repressed, clearly deviating from benchmark protein levels. Hence, the negative feedback model seems worse than no feedback models for predicting experimentally determined benchmark protein levels. In contrast, the calculated final steady state levels for Hrb1p and Ski2p (*n* = 1) are identical to the output of the model with no feedback (at 2543 and 1811 proteins, respectively). In those cases, the parameterisation of the loop ensures that the loop is essentially non-functional. In other words, since *HEK2*, *HRB1* and *SKI2* are commonly predicted to express fewer proteins than benchmark abundance with no-feedback models (Figure 2), the outcome upon applying negative feedback can at best match the performance without feedback. This decreases accuracy as the negative feedback is increased by considering ‘stronger’ feedback dynamics.

Importantly though, increasing the Hill coefficient for *HEK2*, *HRB1* and *SKI2* expression led to further gradual inhibition of expression, while the dissociation constant for protein complex formation had only a minor influence in projected final steady protein levels (Figure 4). However, the effect of the Hill coefficients for the highly abundant Npl3p was different. In this case, closest *NPL3* expression to the benchmark was reached with *n* = 2 and assuming realistic protein dimer formation kinetics (1 μM) (16,454 compared to the benchmark of 47,816 proteins) (Figure 4). As such, the model outperforms the no-feedback model which predicted 104,800 proteins, meaning that the negative feedback model is closer to benchmark by 25,622 proteins in comparison. This arises from the parameterisation where the relatively small measured *K_d_* value (Table 1) has a relatively large reciprocal *K_a_* as a result, which limits the repression of the denominator in Equation (2). This highlights the importance of both parameters when it comes to modelling based on kinetic data.

To evaluate which values of *K_a_* could at best simulate the protein abundance in relation to the *P_ss_*, the feedback models were solved for *K_a_* analytically at the steady state (by quasi-steady state approximation of the mRNA level—Text S1). A positive value could be obtained for *HRB1* and *NPL3* translational autoregulation (dashed green line, Figure 4), while negative values were obtained for *HEK2* and *SKI2*, which are not realistic and therefore not further considered in our analysis. The calculated optimal association kinetics for negative feedback in *HRB1* exactly matched with projected models for the monomeric protein (*n* = 1) considering experimentally determined dissociation constants (Figure 4). For comparison, implementing the optimised positive feedback model for *HRB1* resulted in a better approximation towards the benchmark (3945 proteins at steady state). For *NPL3*, the ‘best-case’ negative dynamics without cooperativity reached the benchmark levels quite accurately, and much better than with *n* = 2 cooperative parameterisation (see above). Conversely, application of optimised positive dynamics overpredicted final protein levels (61,840 proteins) as compared to benchmark levels. Hence, it seemed less accurate as compared to the optimised negative feedback. Overall, these results indicate that by solving the kinetic models, very good approximations to benchmark protein levels could be achieved.

**Figure 5:**
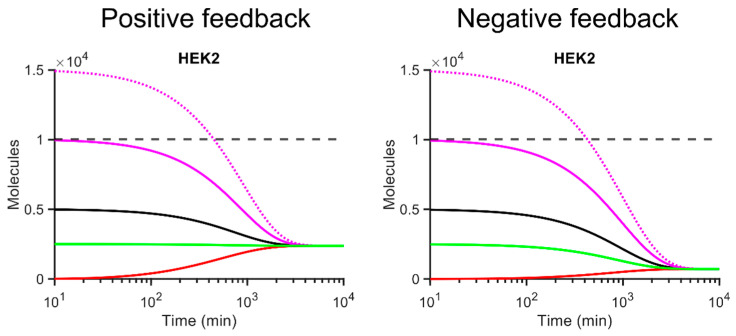
Simulation of autoregulatory gene expression for *HEK2* with different starting concentrations. Positive (left) and negative (right) feedback models (*n* = 1) are parameterised with different initial copy numbers for mRNA (*m_ss_*) and protein (*P_ss_*). Dashed grey line, experimentally determined (benchmark) protein abundance (*P_ss_* = 10,000); solid violet line, *P*_0_ = *P_ss_* and *m*_0_ = *m_ss_*; dashed violet line, *P*_0_ = 1.5 (*P_ss_*) and *m*_0_ = 1.5 (*m_ss_*); black line *P*_0_ = 0.5 (*P_ss_*) and *m*_0_ = 0.5 (*m_ss_*). Green line *P*_0_ = 0.25 (*P_ss_*) and *m*_0_ = 0.25 (*m_ss_*).

We wish to note that numerically determined final steady state protein and mRNA levels are independent of the initial abundance of the repressing RBP, because Equations (2) and (3) have only one non-negative solution at the steady state. This is exemplified for *HEK2* by starting simulations with experimentally determined steady state levels (or fractions thereof) of mRNA and proteins in cells (*P*_0_ = *P_ss_*, *m*_0_ = *m_ss_*) (Figure 5).

### 3.5. Pfk2p and Map1p Protein Levels Are Better Predicted with Positive or Negative Feedback Loops Compared to Models without Feedback

**Figure 6:**
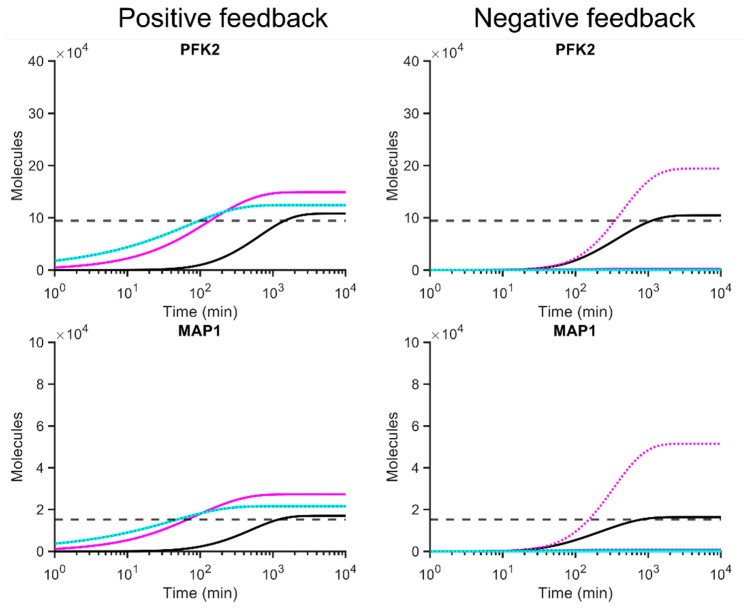
Simulated gene expression with positive/negative translational feedback loops for *PFK2* and *MAP1*, considering the best-case dynamics with predicted values for *K_a_* and *K_d_*. Dashed grey line is the benchmark protein abundance (*P_ss_* Table 1). Solid black line represents each feedback with *n* = 1. Solid violet, *n* = 2 with protein complexing constant of 1 M, dotted violet line, complex formation constant 1 μM. Solid cyan, *n* = 4 with complexing constant of 1 M; dotted cyan line, with complex formation constant 1 μM. Best case dynamics calculated as described in Text S1.

As the RNA binding affinities for Pfk2p and Map1p to RNA are unknown, their *K_a_* and *K_d_* were calculated by solving Equations (2) and (3) with the respective parameters at the steady state, thus predicting ‘best-case’ dynamics (Table 1, Text S1). Considering that Pfk2p and Map1p proteins interact as monomers with their mRNA (Hill coefficient *n* = 1), both positive and negative translational feedback loops predicted protein abundances became very close to the benchmark abundance (Figure 6). In particular, the positive feedback model with *PFK2* predicted 108,100 copies, the negative feedback 104,820 copies and close to experimentally determined benchmark abundance of 94,449 protein copies per cell. For *MAP1*, the positive feedback dynamics predicted 17,027, the negative feedback 16,389 proteins, while the benchmark abundance is 15,179 proteins per cell. Along with modelled steady states for *HRB1* and *NPL3* (Figure 4, dashed green lines), these results substantiate the observation that by solving kinetic models with given data, very good approximations to benchmark protein levels can be achieved.

However, unlike the previously developed feedback models parameterised with known RNA binding affinities, the application of Hill coefficients greater than one resulted in worsening the approximation to benchmark steady state protein levels (Figure 6). With positive feedback models, the predicted steady state abundance of RBPs reached levels above the benchmark. Moreover, faster kinetics were projected to reach steady state levels (Figure 6, left). Negative translational feedback models resulted in over-repressive dynamics (Figure 6, right). Interestingly, an exception to this concerns the Hill coefficient *n* = 2 with a protein complexing constant of 1 μM. This constellation was mildly repressive for *PFK2* and *MAP1* expression. It improved the prediction of cellular Pfk2p abundance (194,120 proteins) towards the benchmark levels (94,449 proteins); and as expected, it showcased mild repressive effect compared to the no-feedback model (371,960 protein, Figure 2). This indicates critical roles for complex formation dynamics associated with Hill coefficients, especially for negative feedback loop models (Figure 4).

## 4. Discussion

Transcriptional feedback loops have been investigated across many organisms, and their relevance for gene expression control is widely recognized. For instance, they are responsible for the occurrence of multistable behaviour, and possible oscillatory dynamics [15,47]. In such studies, a set of parameters get associated to external factors and assumed to vary over time or according to external conditions. By exceeding certain threshold values, the parameters drive the network through a mathematical bifurcation, which can describe the transition from monostable to multistable or oscillatory dynamics. Furthermore, transcriptional feedback loops have also been extended to complex topological scenarios, leading to rich dynamical behaviours. Interlocked feedback loops, for instance, have been shown to be relevant in bacterial quorum sensing mechanisms, while variants of the repressilator were incorporated into model cell differentiation [48,49]. Noise control and propagation has also been largely investigated in both negative and positive feedback loops [50,51]. Overall, it has now become clear that transcriptional feedback is an essential feature of many regulatory processes.

In contrast, little is known about the importance of feedback loops at the post-transcriptional level, and for translational regulation in particular [52]. In this study, we have framed and investigated translational feedback loops for putative autoregulatory RBPs, and assessed their relevance for establishing experimentally determined steady state protein levels in cells. Besides the projection of no-feedback models for comparison, positive and negative translational feedback loops were simulated using experimentally determined parameters for binding of putative RBPs to their own mRNA. Furthermore, hypothetical cooperative binding situations through Hill coefficients involving complex formation kinetics for dimers or tetramers were considered and explored to give a simple sensitivity analysis.

We have preferred to use available empirical knowledge as much as possible to inform an exploration of post-transcriptional feedback topologies. For example, when exploring Hill coefficients (which are largely unknown) we have continued to use known values for *K_d_* and *K_a_*. Similarly, both positive and negative feedback topologies described here act by modulation of the translation rate which also considers the binding dynamics and protein abundance. As *k*_3_ is taken from a study where the state of feedback and mRNA abundance is not strictly known, it is not certain whether this figure is from a cell with either active or inactive post-transcriptional feedback. We have thus taken the parameter value of *k*_3_ throughout to be the rate constant in our calculations.

We also note that when evaluating more complex models such as the feedback models vs. a simpler one (without feedback), increasing the number of parameters in a model will improve its performance relative to our benchmark. Therefore, we have introduced as few “new” parameters as possible, in terms of association dynamics and the Hill coefficient, which are evidence-based or express realistic biochemical functions. In this regard, the Hill coefficient and associated cooperativity could be relevant for some proteins, such as Pfk2p that forms a heterooctameric complex with Pfk1p in *S. cerevisiae* [29].

Generally, protein expression modelled with feedback led to closer approximations towards experimentally defined benchmark protein levels, as compared to simple no-feedback models. This was particularly the case using positive feedback loops, while negative feedback loops were rather repressive, applying realistic RBP-RNA dynamics, although they could still be more accurate than simple models without feedback overpredicting protein expression. In the case of Npl3p, we found that cooperative binding of a dimer formed with a realistic dimerisation constant (1 μM) could be used to fine tune negative feedback of simple models which otherwise overpredict the protein expression. This provides a glimpse of the more nuanced form that negative post-transcriptional feedback may take compared to positive feedback. Finally, we found that calculated best case scenarios can provide very good approximation towards benchmarked protein steady state levels. Intriguingly, the calculated *K_a_* for Hrb1p derived from kinetic data was *quasi*-identical to the experimentally determined constant (Table 1). Although likely a coincidence, this result indicates that, in principle, relevant association constants can be obtained by solving the parametrised equations proposed herein, an observation that is remarkable considering the many limitations and inaccuracies that the underlying experimental data may contain.

For instance, the steady state protein levels used as benchmarks in this study may truly be the result of a series of ‘bursty’ expression events that average en masse [53]. Any single cell may deviate significantly from the average abundances, as a product of intrinsic/extrinsic noise. Such dynamics could give rise to multistable behaviour, where switching between one steady state to another may occur [54]. However, multistable or oscillatory behaviour is precluded here due to the steady state assumed in this analysis along with the exclusion of any extrinsic/intrinsic sources of noise. Furthermore, the experimental data (Table 1) used for modelling assumes compatibility between the various growth/experimental conditions employed. While most data were obtained from cells grown in YPD media, save for *k*_3_, protein synthesis, cells were grown in SC medium. While both YPD and SC media are rich in nutrients, cells grow slower in SC media, implying reduced protein synthesis rates [55]. Additionally, the acquisition of mRNA degradation rates (*k*_2_) involved a temperature shift of cells from 24 °C to 37 °C, which may imply a heat shock response affecting mRNA stability [56,57]. Finally, our models concern exponential (log) phase of growth which may differ greatly for lag, stationary, or death phases where different expression regimes come into play. Certain feedback loops may only become active in one of those growth-phases or at a specific cell-cycle stages. In regard to the simple autoregulatory feedbacks considered here, such stage-specific control could be implemented through post-translational modification of the RBP affecting its stability, complex formation and RNA-binding affinity, as well as the interaction with other cellular components.

Increasing the Hill coefficient *n* of the modelled feedback interaction assumes that the RBP forms multimers, each cooperatively increasing the affinity for the mRNA. However, other arrangements may also be considered, such as individual RBP interacting with the mRNA first and subsequent binding of additional RBPs. As such, the effect of alternative binding arrangements remains to be further explored, as well as the inclusion of additional regulatory elements, such as function-modulation by RNA folding and competition/synergistic effects through second RBPs and/or non-coding RNAs [58]. Regarding the latter, there is growing evidence for a multitude of RNA functions, including acting as aptamers, ribozymes, or regulatory platforms themselves for other coupled processes by interaction with ncRNAs and other RBPs [59]. Indeed, the folding of mRNA can represent labile mRNA forms which are sterically sequestered to the initiation complex eIF4F [60]. These diverse activities may make the mRNA unavailable for translation, forming several reversibly inactive pools of mRNA governed by additional rate constants. The un-modelled lifespan of mRNA-protein complexes may also create further interplay in the true dynamics where long-lived RNPs act as a store of either particle with independent association kinetics. Not considered here are also alternative feedback architectures, such as transcriptional feedback (i.e., the RBP acting a transcription factor), which could also contribute to differences seen between the protein expression predictions under translation feedback and the known abundances.

## 5. Conclusions

Our study exemplified that simulated protein abundance values could in principle approximate experimentally determined abundances solely by adjusting the translation rate. We found that modelling of positive translational feedback loops led to best approximations and are sensitive to changes in the Hill coefficients. Conversely, negative translational feedback loops seemed to be highly repressive, except for Npl3p dimers with the assumed dimerisation of 1 μM. In this regard, the interaction of homo-monomers, dimers and tetramers was simulated for each RBP, and two kinetic regimes for the formation of the RNA-binding protein dimers were tested. The latter was necessary as stoichiometries and kinetics are not yet widely documented for RBPs, especially for those that may regulate their own translation. Remarkably, we found that integration of fully calculated RNA association constants for RBPs by considering the available kinetic data can, in principle, lead to very good approximations of benchmarked protein steady state levels.

It should be emphasised that our simulations cannot represent proof for the presence of feedback loops. Applied to putative autoregulatory RBPs, the models should be used only for prediction of the characteristics of suspected feedback interactions. Nevertheless, the topologies of post-transcriptional feedback certainly warrant further investigation. For instance, autoregulatory feedback dynamics could have evolved as pacemakers for critical cell functions. The degree of self-regulation in turn could also affect the noise of each of these components and its contribution to gene expression. The combination of data and modelling would enhance our understanding of the link between evolutionary decision making and the pervasive molecular decision making of microbes.

## Figures and Tables

**Table 1:** Parameters for modelling potential autoregulatory RBPs. The parameter *k*_1_ is the transcription rate; *k*_2_, the mRNA degradation constant; *k*_3_, the translation rate constant; *k*_4_, the protein degradation constant; *P_ss_*, the mean protein abundance (steady state assumed); *m_ss_*, the mean mRNA abundance (steady state assumption). Notice that *k*_1_ has units molecules min^−1^; *k*_2–4_ have units min^−1^; protein and mRNA abundances are given in numbers of molecules. *K_d_* is given as molecules cell^−1^ whereas *K_a_* is given as cells molecule^−1^. * Predicted values (Text S1).

Parameter	*HRB1*	*HEK2*	*SKI2*	*NPL3*	*PFK2*	*MAP1*
*k*_1_	1.08 × 10^−1^	1.97 × 10^−1^	1.35 × 10^−1^	3.26 × 10^−1^	5.79 × 10^−1^	4.90 × 10^−1^
*k*_2_	7.82 × 10^−2^	8.19 × 10^−2^	3.01 × 10^−2^	2.46 × 10^−2^	2.26 × 10^−2^	3.70 × 10^−2^
*k*_3_	1.13	3.99 × 10^−1^	4.97 × 10^−1^	9.23	1.54 × 10	1.03 × 10
*k*_4_	6.10 × 10^−4^	9.66 × 10^−4^	1.23 × 10^−3^	1.16 × 10^−3^	1.06 × 10^−3^	1.55 × 10^−3^
*P_ss_*	4.79 × 10^3^	1.00 × 10^4^	4.62 × 10^3^	4.78 × 10^4^	9.44 × 10^4^	1.52 × 10^4^
*m_ss_*	3.00	8.55	1.37 × 10	5.10 × 10^−1^	2.15 × 10	1.15 × 10
*K_d_*	6.05 × 10^4^	1.8 × 10^3^	1.01 × 10^4^	5.04 × 10	2.17 × 10^5^ *	6.14 × 10^4^ *
*K_a_*	1.65 × 10^−5^ 1.65 × 10^−5^ *	5.56 × 10^−4^	9.90 × 10^−5^	1.98 × 10^−2^ 2.60 × 10^−5^ *	2.43 × 10^−5^ *	2.67 × 10^−4^ *

## Data Availability

The data presented in this study are available in Supplementary Materials Table S1 and Text S1.

## Supplementary Materials

The following are available online at https://www.mdpi.com/article/10.3390/microorganisms10020340/s1, Table S1: Parameters assembled for 5854 yeast proteins; Text S1: Calculation of best-case predicted feedback dynamics.
