# Supplementary material for: Parameterising Translational Feedback Models of Autoregulatory RNA-Binding Proteins in Saccharomyces cerevisiae
Source: Microorganisms. 2022 Feb 1;10(2):340. doi: 10.3390/microorganisms10020340 (PMC8874390; doi:10.3390/microorganisms10020340)
Supplement: Supplementary file 1 [file microorganisms-10-00340-s001.zip › microorganisms-1522418-supplementary.pdf]

Supplementary Materials

Parameterising Translational Feedback Models of Autoregulatory RNA-Binding Proteins in *Saccharomyces cerevisiae*

Michael Clarke-Whittet [1,2], Andrea Rocco [2,3,*] and André P. Gerber [2,*]

Text S1: Calculation of best-case predicted feedback dynamics.

Using the equations when $n = 1$ (negative feedback):

$$\begin{cases} \dfrac{dm}{dt} = k_1 - k_2 m \\ \dfrac{dP}{dt} = \dfrac{k_3 m}{1 + K_a P} - k_4 P \end{cases} \quad (1)$$

At the steady state:

$$\begin{cases} \dfrac{dm_{ss}}{dt} = 0 \\ \dfrac{dP_{ss}}{dt} = 0 \end{cases} \quad (2)$$

$$\begin{cases} m_{ss} = \dfrac{k_1}{k_2} \\ k_3 m_{ss} - k_4 P_{ss} - K_a k_4 {P_{ss}}^2 = 0 \end{cases} \quad (3)$$

$$K_a = \frac{k_3 m_{ss} - k_4 P_{ss}}{k_4 {P_{ss}}^2} \quad (4)$$

And likewise, for positive feedback when $n = 1$:

$$\begin{cases} \dfrac{dm}{dt} = k_1 - k_2 m \\ \dfrac{dP}{dt} = \dfrac{k_3 m P}{K_d + P} - k_4 P \end{cases} \quad (5)$$

So that again,

$$\begin{cases} m_{ss} = \dfrac{k_1}{k_2} \\ k_3 m P - K_d k_4 P_{ss} + k_4 {P_{ss}}^2 = 0 \end{cases} \quad (6)$$

$$\begin{cases} m_{ss} = \dfrac{k_1}{k_2} \\ K_d = \dfrac{k_3 m P - k_4 {P_{ss}}^2}{k_4 P_{ss}} \end{cases} \quad (7)$$
